# Diabetes-duration-related shifts in inflammation-resolving lipid mediator signatures and their association with 3-month functional outcome in large artery atherosclerotic stroke

**DOI:** 10.3389/fneur.2026.1841577

**Published:** 2026-07-01

**Authors:** Wei-Meng Guo, Dong-Liang Tian, Zhong-Xu Gao, Hai-Chao Lv, Shuo Sun

**Affiliations:** 1 Department of Neurology, Affiliated Hospital of Hebei University, Clinical Medical College, Hebei University, Baoding, Hebei, China; 2 Department of Neurosurgery, Baoding First Central Hospital, Baoding, Hebei, China; 3 Department of Psychiatry, Hebei Provincial Mental Health Center, The Sixth Clinical Medical College Hebei University, Baoding, Hebei, China; 4 Department of Neurosurgery, Affiliated Hospital of Hebei University, Clinical Medical College, Hebei University, Baoding, Hebei, China

**Keywords:** functional outcome, large artery atherosclerosis stroke, lipoxin A4, machine learning, specialized pro-resolving lipid mediators, type 2 diabetes mellitus

## Abstract

**Background:**

Specialized pro-resolving lipid mediators are involved in post-stroke neuroinflammation, but their relevance in large artery atherosclerotic (LAA) stroke, particularly in relation to diabetes duration, remains unclear.

**Methods:**

In this single-center retrospective study, 175 patients with LAA stroke were enrolled. Serum lipoxin A4 (LXA4), leukotriene B4 (LTB4), and resolvin D2 (RvD2) were measured within 72 h of stroke onset. Functional outcome at 3 months was assessed using the modified Rankin Scale. Analyses were stratified by type 2 diabetes mellitus (T2DM) status and diabetes duration (<5 vs. ≥5 years). Logistic regression and linear support vector machine models were used in exploratory analyses.

**Results:**

Of the 175 patients, 130 had a favorable outcome and 45 a poor outcome. LXA4 levels were higher in the favorable-outcome group, although the difference was not significant. No significant differences in LXA4, LTB4, or RvD2 were observed between patients with and without T2DM. In contrast, within the T2DM subgroup, patients with a diabetes duration of ≥5 years showed higher LXA4 and LTB4 levels and a lower RvD2/LTB4 ratio than those with a duration of <5 years. Diabetes duration correlated positively with both LXA4 and LTB4. In exploratory machine learning analyses, LXA4 was consistently retained, and model performance declined after its removal.

**Conclusion:**

These findings are exploratory. LAA stroke was associated with diabetes duration-related remodeling of specialized pro-resolving lipid mediator profiles. Compared with diabetes status alone, diabetes duration may better reflect metabolic-inflammatory heterogeneity. These findings are exploratory; LXA4 did not demonstrate a statistically significant association with functional outcome, and no independent prognostic value has been established. LXA4 may represent a candidate signaling molecule warranting further investigation.

## 1 Introduction

Ischemic stroke (IS) remains one of the leading neurological diseases worldwide in terms of mortality and long-term disability. According to the TOAST etiological classification, large artery atherosclerosis (LAA) is one of the major etiological subtypes of IS, accounting for approximately 20% of all ischemic strokes (1). In patients with LAA stroke, neuroinflammatory cascades triggered by ischemia–reperfusion injury, blood–brain barrier (BBB) disruption, and secondary neuronal death and dysfunction are closely related to clinical outcome. These processes are jointly shaped by the dynamic balance between pro-inflammatory and pro-resolving pathways (2, 3). Therefore, identifying biological signals associated with stroke outcome from the perspective of inflammation-regulatory networks may provide information beyond that offered by traditional vascular risk factors and conventional clinical stratification markers.

Type 2 diabetes mellitus (T2DM) is one of the most important metabolic comorbidities of ischemic stroke (4). Compared with individuals without diabetes, patients with diabetes have an approximately 1.5- to 2-fold higher risk of stroke, and this risk may further increase with longer diabetes duration (5). Chronic hyperglycemia may accelerate atherosclerotic progression and amplify neuroinflammatory responses in diabetic ischemic stroke through multiple mechanisms, including endothelial dysfunction, oxidative stress, persistent activation of the AGE-RAGE signaling axis, and the establishment of a pro-inflammatory and pro-thrombotic microenvironment (6–8). In addition, T2DM is often accompanied by elevated homocysteine (Hcy) levels, which may further aggravate endothelial injury and accelerate atherosclerosis, thereby increasing the risk of unfavorable stroke outcomes (9). Previous studies have mainly focused on the impact of the presence or absence of diabetes on stroke outcome. However, compared with such a binary classification, diabetes duration may more directly influence the status of post-stroke inflammation-regulatory networks. Clinical evidence addressing this issue, however, remains limited. Recent clinical studies have further highlighted the role of inflammatory resolution pathways and metabolic comorbidities in stroke prognosis and post-stroke inflammatory heterogeneity (10–13).

In recent years, the resolution of inflammation has come to be recognized not as a passive termination of the inflammatory response, but as an active and highly coordinated process governed by endogenous bioactive programs, in which specialized pro-resolving mediators (SPMs) serve as key regulatory molecules (10). Lipoxin A4 (LXA4) and resolvin D2 (RvD2) are representative pro-resolving mediators, whereas leukotriene B4 (LTB4) is an important pro-inflammatory lipid mediator (14). Experimental studies have shown that LXA4 and related pathways can alleviate post-ischemic inflammatory injury, BBB disruption, and neurological dysfunction (15). Clinical studies have also suggested that lower LXA4 levels and a reduced RvD2/LTB4 ratio are associated with poor outcome after stroke, and that impaired inflammatory resolution may be even more pronounced in the setting of diabetes (16, 17). These findings suggest that the evolution of stroke outcome may depend not simply on the absolute level of a single lipid mediator, but rather on the relative balance between pro-inflammatory and pro-resolving mediators and on the overall pattern of lipid mediator profile changes.

Nevertheless, current clinical studies still have three major limitations. First,most have focused on isolated analyses of single mediators, while integrated investigations of specialized pro-resolving lipid mediator profiles in patients with LAA stroke remain lacking. Second, insufficient attention has been paid to the effect of diabetes duration, rather than the mere presence or absence of diabetes, on the regulation of lipid mediators. Third, prognostic analyses have largely remained restricted to traditional univariable approaches or conventional regression models, and the contribution of multidimensional biomarkers within combined analytical frameworks has not been adequately assessed. Accordingly, the present study focused on patients with LAA stroke and investigated diabetes duration-related remodeling of specialized pro-resolving lipid mediator profiles. We further examined the association between LXA4 and 3-month functional outcome and evaluated its potential indicative value within a multivariable framework by combining stratified analyses with machine learning approaches.

## 2 Materials and methods

### 2.1 Study design and participants

This was a single-center retrospective study. We enrolled 175 consecutive patients with large artery atherosclerosis (LAA) stroke who were admitted to the Department of Neurology, Affiliated Hospital of Hebei University, between December 2024 and December 2025. Acute ischemic stroke was diagnosed according to the Chinese Guidelines for the Diagnosis and Treatment of Acute Ischemic Stroke 2023, based on clinical presentation and neuroimaging findings on cranial computed tomography (CT) or magnetic resonance imaging (MRI), including diffusion-weighted imaging (DWI) and apparent diffusion coefficient (ADC) sequences. The LAA subtype was determined on the basis of head-and-neck computed tomography angiography (CTA) or magnetic resonance angiography (MRA) findings and was independently confirmed by two board-certified neurologists.

The inclusion criteria were as follows: age ≥ 18 years; blood sampling completed within 72 h of stroke onset; fulfillment of the diagnostic criteria for LAA stroke; and, for patients with type 2 diabetes mellitus (T2DM), fulfillment of the diagnostic criteria defined in the Chinese Guidelines for the Prevention and Treatment of Type 2 Diabetes Mellitus (2020 edition).

The exclusion criteria were as follows: cardioembolic stroke, stroke of other determined etiology, or stroke of undetermined etiology; concomitant angina pectoris or myocardial infarction; severe hepatic or renal dysfunction; psychiatric disorders; coma; other endocrine diseases; malignancy or autoimmune disease; acute systemic inflammation within 1 month; receipt of thrombolytic therapy; substantial pre-stroke disability [modified Rankin Scale (mRS) score ≥ 3]; random blood glucose ≥ 16.7 mmol/L or ≤ 3.9 mmol/L before admission; and any other condition that might influence inflammatory markers or outcome assessment.

The study was approved by the Ethics Committee of the Affiliated Hospital of Hebei University.

### 2.2 Biomarker measurements and clinical data collection

Venous blood samples were obtained from all enrolled patients within 72 h of stroke onset. After clotting at room temperature for 30 min, samples were centrifuged at 2000 × g for 20 min, and the separated serum was stored at −20 °C until analysis. Serum levels of LXA4, leukotriene B4 (LTB4), and resolvin D2 (RvD2) were quantified using enzyme-linked immunosorbent assay (ELISA), and the RvD2/LTB4 ratio was subsequently calculated.

Inflammation-related parameters were collected simultaneously, including the systemic immune-inflammation index (SII), systemic inflammation response index (SIRI), platelet-to-lymphocyte ratio (PLR), lymphocyte-to-monocyte ratio (LMR), fibrinogen, and homocysteine (Hcy). Baseline clinical variables were also recorded, including age, sex, infarct location, hypertension, smoking history, alcohol consumption, previous cerebral infarction, and diabetes status.

### 2.3 Outcome assessment and stratification

The primary outcome was functional outcome at 3 months after stroke onset, assessed using the modified Rankin Scale (mRS). A score of 0–2 was defined as a good functional outcome, whereas a score of 3–6 was defined as a poor functional outcome. The 3-month mRS score, assessed at outpatient follow-up, was used as the primary outcome variable in all analyses. Supplementary materials, including variable labels and units, are provided in English for transparency and reproducibility.

In line with the study objectives, analyses were performed within a predefined stratification framework. First, patients were classified into good- and poor-outcome groups according to the 3-month mRS score to compare clinical characteristics and lipid mediator profiles between patients with different functional outcomes. Second, patients were stratified by diabetes status into a non-T2DM group and a T2DM group to examine the association between diabetes and lipid mediator profiles. Third, within the T2DM subgroup, patients were further divided into a duration of < 5 years group and a duration of ≥ 5 years group to explore the impact of diabetes duration on specialized pro-resolving lipid mediator profiles.

Previous studies of T2DM have commonly used disease duration for subgroup stratification, with 5 years serving as one of the frequently adopted cut-off values. In addition, longer diabetes duration has been associated with a higher risk of ischemic stroke and other major macrovascular events. On the basis of this literature background, and given the limited sample size of the diabetic subgroup as well as the relatively balanced subgroup sizes achieved with a 5-year threshold, we used < 5 years and ≥ 5 years as an exploratory stratification scheme in the present study. This cutoff is acknowledged as arbitrary and exploratory; the choice was informed by literature precedent and practical considerations of subgroup balance rather than a validated clinical threshold (18–20).

The 3-month mRS score was obtained at outpatient follow-up and assessed by trained investigators using a standardized protocol. Outcome assessors were blinded to baseline lipid mediator measurements. All patients included in the final analysis completed the 3-month follow-up assessment.

### 2.4 Statistical analysis and machine learning

The distribution of continuous variables was first assessed using the Shapiro–Wilk test. Variables with a normal distribution are presented as mean ± standard deviation (SD) and were compared between groups using the independent-samples *t*-test. Variables with a non-normal distribution are presented as median (interquartile range [IQR]) and were compared using the Wilcoxon rank-sum test. Categorical variables are presented as number (percentage) and were compared using the chi-square test or Fisher’s exact test, as appropriate. Correlations were assessed using Spearman’s rank correlation analysis. All statistical tests were two-sided, and a *p* value < 0.05 was considered statistically significant. Prior to multivariable logistic regression modelling, multicollinearity among candidate predictors was assessed using the variance inflation factor (VIF). Variables with VIF > 10 were considered to indicate substantial multicollinearity and were handled by exclusion or combination. As the primary outcome was binary (good vs. poor 3-month functional status), logistic regression rather than Cox proportional hazards regression was the primary modelling approach; the proportional hazards assumption was therefore not applicable. For descriptive purposes, the distribution of each predictor was examined across outcome groups prior to model construction to inform variable selection.

To further explore the association between lipid mediators and 3-month functional outcome in a multivariable setting, machine learning methods were applied in an exploratory analysis. The dataset was randomly divided into training and test sets in a 7:3 ratio using stratified sampling to maintain an approximately balanced distribution of outcome categories across the two sets. All machine learning analyses were implemented in Python 3.11 using the scikit-learn library (version 1.4.2). Logistic regression (LR) and linear-kernel support vector machine (SVM) models were then constructed. For the LR model, feature selection was performed using recursive feature elimination with cross-validation (RFECV). RFECV used logistic regression as the base estimator with 3-fold stratified cross-validation (StratifiedKFold, cv. = 3) to evaluate each feature subset, with AUC as the scoring metric, and a minimum of one feature retained. The final logistic regression model was trained with the following parameters: penalty = ‘l2’, solver = ‘liblinear’, max_iter = 1,000, random_state = 42. For the SVM model, feature selection was performed using recursive feature elimination (RFE) with a linear-kernel SVM (kernel = ‘linear’, C = 1.0, random_state = 42) as the base estimator. The dataset split used random_state = 42 to ensure reproducibility. In addition, ablation analysis was used to assess the relative informational contribution of key features to model performance.

Model performance was evaluated using the area under the receiver operating characteristic curve (AUC), sensitivity, specificity, and accuracy. For the LR model, the F1 score and Matthews correlation coefficient (MCC) were additionally reported. Ninety-five percent confidence intervals (CIs) for the AUC were estimated by bootstrap resampling with 1,000 iterations. Given the limited sample size and the lack of independent external validation, the machine learning findings were interpreted as exploratory evidence regarding the informational value of candidate features in a multivariable framework, rather than as the basis for a clinically generalizable prediction tool.

Regarding missing data, all 175 enrolled patients completed the 3-month follow-up assessment, so there were no missing values for the primary outcome variable. For the key biomarker and clinical variables analyzed, missing data were minimal (fewer than 5 values missing for any single variable). A complete-case analysis approach was adopted; cases with missing covariate values were excluded from the specific analyses in which those variables were required. The number of missing observations for each variable is reported in Supplementary materials.

## 3 Results

### 3.1 Comparison according to functional outcome

A total of 175 patients with LAA stroke were included in the analysis, of whom 130 (74.3%) had a good functional outcome and 45 (25.7%) had a poor functional outcome.

Among continuous variables, serum LXA4 levels were higher in the good-outcome group than in the poor-outcome group, although the difference did not reach statistical significance [104.43 (86.35–126.03) pg./mL vs. 96.04 (84.30–109.87) pg./mL, *p* = 0.097]. No significant between-group differences were observed in LTB4 or RvD2 levels (both *p* > 0.05). Likewise, total cholesterol, low-density lipoprotein cholesterol (LDL-C), homocysteine (Hcy), the systemic immune-inflammation index (SII), and the systemic inflammation response index (SIRI) did not differ significantly between the two groups (all *p* > 0.05).

Among categorical variables, sex distribution was the only baseline characteristic that differed significantly between the two groups (*p* = 0.038). The proportion of female patients was higher in the poor-outcome group than in the good-outcome group [51.1% (23/45) vs. 32.3% (42/130)]. No significant differences were found between the two groups with respect to age, infarct location, hypertension, history of cerebral infarction, alcohol consumption, or smoking status (all *p* > 0.05). In addition, the prevalence of diabetes was higher in the good-outcome group than in the poor-outcome group [33.8% (44/130) vs. 17.8% (8/45)], although this difference did not reach statistical significance (*p* = 0.065). Detailed data are presented in Table 1.

**Table 1:** Baseline characteristics according to 3-month functional outcome in patients with LAA stroke (main variables).

Variable	Category	Good outcome (*n* = 130)	Poor outcome (*n* = 45)	*p* value	Statistical test
LXA4 (pg/mL)	—	104.43 (86.35 ~ 126.03)	96.04 (84.30 ~ 109.87)	0.097	Wilcoxon rank-sum
LTB4 (pg/mL)	—	55.26 (45.81 ~ 64.87)	52.36 (46.26 ~ 60.52)	0.196	Wilcoxon rank-sum
Total cholesterol (mmol/L)	—	4.26 ± 0.93	4.30 ± 1.01	0.817	Independent-samples t
LDL-C (mmol/L)	—	2.53 ± 0.73	2.52 ± 0.83	0.935	Independent-samples t
Homocysteine (μmol/L)	—	13.00 (11.00 ~ 16.00)	12.00 (11.00 ~ 17.00)	0.990	Wilcoxon rank-sum
SII	—	698.39 (473.03 ~ 1108.85)	761.72 (497.64 ~ 1044.07)	0.471	Wilcoxon rank-sum
SIRI	—	1.46 (0.96 ~ 2.06)	1.46 (0.97 ~ 2.78)	0.460	Wilcoxon rank-sum
Sex	Female	42 (32.3%)	23 (51.1%)	0.038	Chi-square
	Male	88 (67.7%)	22 (48.9%)		
Age ≥ 65 years	Yes	61 (46.9%)	26 (57.8%)	0.279	Chi-square
Hypertension	Yes	91 (70.0%)	34 (75.6%)	0.603	Chi-square
History of cerebral infarction	Yes	40 (30.8%)	20 (44.4%)	0.138	Chi-square
Diabetes mellitus	Yes	44 (33.8%)	8 (17.8%)	0.065	Chi-square

Normally distributed continuous variables are presented as mean ± standard deviation (SD) and were compared using the independent-samples t test. Non-normally distributed continuous variables are presented as median (interquartile range [IQR]) and were compared using the Wilcoxon rank-sum test. Categorical variables are presented as number (percentage) and were compared using the chi-square test or Fisher’s exact test, as appropriate.

### 3.2 Comparison by T2DM status

Of the 175 patients included, 123 were in the non-T2DM group and 52 were in the T2DM group. Stratification by T2DM status showed no significant differences in LXA4, LTB4, or RvD2 levels between the two groups (all *p* > 0.05). However, plasma fibrinogen levels were lower in the T2DM group than in the non-T2DM group [3.23 (2.80–3.41) g/L vs. 3.37 (2.93–3.82) g/L, *p* = 0.035], whereas homocysteine (Hcy) levels were higher [14.00 (11.90–18.25) μmol/L vs. 12.00 (10.25–15.00) μmol/L, *p* = 0.005].

Among categorical variables, patients aged ≥ 65 years accounted for a smaller proportion of the T2DM group than of the non-T2DM group [32.7% (17/52) vs. 56.9% (70/123), *p* = 0.006], while a history of smoking was more common in the T2DM group [86.5% (45/52) vs. 18.7% (23/123), *p* < 0.001]. Sex distribution did not differ significantly between the two groups (*p* = 0.56). The proportion of patients with a poor 3-month functional outcome was lower in the T2DM group than in the non-T2DM group [15.4% (8/52) vs. 30.1% (37/123)], although this difference did not reach statistical significance (*p* = 0.065). The detailed results are shown in Table 2.

**Table 2:** Baseline characteristics according to diabetes status in patients with LAA stroke.

Variable	Category	Non-T2DM (n = 123)	T2DM (n = 52)	*p* value	Statistical test
LXA4 (pg/mL)	—	101.29(85.89 ~ 120.01)	98.95(81.71 ~ 113.59)	0.467	Wilcoxon rank-sum
LTB4 (pg/mL)	—	55.90(46.33 ~ 62.61)	52.45(45.30 ~ 61.81)	0.298	Wilcoxon rank-sum
Plasma fibrinogen (g/L)	—	3.37(2.93 ~ 3.82)	3.23(2.80 ~ 3.41)	0.035	Wilcoxon rank-sum
Homocysteine (μmol/L)	—	12.00(10.25 ~ 15.00)	14.00(11.90 ~ 18.25)	0.005	Wilcoxon rank-sum
Sex	Female	44(35.8%)	21(40.4%)	0.56	Chi-square
	Male	79(64.2%)	31(59.6%)		
Age (≥ 65 years)	Yes	70(56.9%)	17(32.7%)	0.006	Chi-square
Smoking history	Yes	23(18.7%)	45(86.5%)	<0.001	Chi-square
Poor 3-month functional outcome (mRS)	Yes	37(30.1%)	8(15.4%)	0.065	Chi-square

As in Table 1. *p* < 0.05 was considered statistically significant.

### 3.3 Comparison by diabetes duration among patients with T2DM

Among the 52 patients with T2DM, 27 had a diabetes duration of < 5 years and 25 had a duration of ≥ 5 years. No significant between-group differences were found in age, infarct location, alcohol consumption, history of cerebral infarction, hypertension, or 3-month functional outcome (all *p* > 0.05). Poor functional outcome occurred in 18.5% (5/27) of patients in the < 5 years group and 12.0% (3/25) of those in the ≥ 5 years group, with no significant difference between the groups (*p* = 0.705).

Marked differences were observed in lipid mediator profiles according to diabetes duration. Patients with a diabetes duration of ≥ 5 years had significantly higher levels of both LXA4 and LTB4 than those with a duration of < 5 years [LXA4: 114.00 (110.15–143.53) pg./mL vs. 81.75 (71.10–91.57) pg./mL, *p* < 0.001; LTB4: 56.05 (48.94–65.36) pg./mL vs. 47.19 (40.23–57.70) pg./mL, *p* = 0.016]. By contrast, the RvD2/LTB4 ratio was significantly lower in the ≥ 5 years group than in the < 5 years group [1.59 (1.45–1.93) vs. 1.97 (1.79–2.16), *p* = 0.013]. RvD2 levels did not differ significantly between the two groups (*p* = 0.845).

Among the inflammation-related indices, SIRI was higher in patients with a diabetes duration of < 5 years than in those with a duration of ≥ 5 years [1.62 (1.30–2.23) vs. 1.24 (0.79–1.79), *p* = 0.048], whereas LMR was higher in the ≥ 5 years group [3.73 (3.13–4.53) vs. 3.00 (2.45–3.43), *p* = 0.009]. No significant between-group differences were observed in SII, PLR, or Hcy (all *p* > 0.05). Detailed results are presented in Table 3.

**Table 3:** Baseline characteristics according to diabetes duration in patients with T2DM (main variables).

Variable	Category	Diabetes duration < 5 years (*n* = 27)	Diabetes duration ≥ 5 years (*n* = 25)	*p* value	Statistical test
LXA4 (pg/mL)	—	81.75(71.10 ~ 91.57)	114.00(110.15 ~ 143.53)	<0.001	Wilcoxon rank-sum
LTB4 (pg/mL)	—	47.19(40.23 ~ 57.70)	56.05(48.94 ~ 65.36)	0.016	Wilcoxon rank-sum
RvD2/LTB4	—	1.97(1.79 ~ 2.16)	1.59(1.45 ~ 1.93)	0.013	Wilcoxon rank-sum
SIRI	—	1.62(1.30 ~ 2.23)	1.24(0.79 ~ 1.79)	0.048	Wilcoxon rank-sum
LMR	—	3.00(2.45 ~ 3.43)	3.73(3.13 ~ 4.53)	0.009	Wilcoxon rank-sum
RvD2 (pg/mL)	—	94.98 ± 13.90	95.86 ± 18.40	0.845	Independent-samples t
Homocysteine (μmol/L)	—	14.00(12.90 ~ 19.50)	14.00(11.00 ~ 18.00)	0.413	Wilcoxon
Poor 3-month functional outcome (mRS)	Yes	5(18.5%)	3(12.0%)	0.705	Fisher’s exact

### 3.4 Correlations of diabetes duration with lipid mediators in patients with T2DM

Spearman correlation analysis in the T2DM subgroup showed that diabetes duration was strongly positively associated with LXA4 levels (rs = 0.866, *p* < 0.001) and was also positively associated with LTB4 levels (rs = 0.337, *p* = 0.014). The RvD2/LTB4 ratio was inversely correlated with both LXA4 (rs = −0.360, *p* = 0.009) and LTB4 (rs = −0.691, *p* < 0.001). In addition, RvD2 was positively correlated with LTB4 (rs = 0.295, *p* = 0.033), and LMR was likewise positively correlated with LTB4 (rs = 0.293, *p* = 0.035). By contrast, SIRI showed an inverse but non-significant association with LTB4 (rs = −0.238, *p* = 0.090). No significant correlations were identified between LXA4 or LTB4 and the remaining clinical or inflammatory variables. The detailed results are summarized in Table 4.

**Table 4:** Spearman correlations of clinical and inflammatory variables with serum LXA4 and LTB4 levels in patients with T2DM.

Variable	LXA4, rs	*p* value	LTB4, rs	*p* value
RvD2 (pg/mL)	−0.021	0.883	0.295	0.033
RvD2/LTB4	−0.360	0.009	−0.691	<0.001
BMI (kg/m^2^)	0.198	0.159	0.135	0.339
Plasma fibrinogen (g/L)	−0.143	0.312	−0.146	0.303
Total cholesterol (mmol/L)	0.178	0.208	−0.097	0.492
Triglycerides (mmol/L)	0.192	0.173	−0.023	0.873
LDL-C (mmol/L)	0.168	0.233	−0.116	0.413
Homocysteine (μmol/L)	−0.014	0.920	−0.219	0.118
SII	0.161	0.260	−0.107	0.453
PLR	−0.177	0.209	−0.037	0.796
SIRI	−0.087	0.541	−0.238	0.090
LMR	0.201	0.152	0.293	0.035
Diabetes duration	0.866	<0.001	0.337	0.014

rs, Spearman rank correlation coefficient. Because all variables were non-normally distributed according to the Shapiro–Wilk test, correlations were assessed using Spearman rank correlation analysis. Bold values indicate statistical significance (*p* < 0.05). Abbreviations: LXA4, lipoxin A4; LTB4, leukotriene B4; RvD2, resolvin D2; BMI, body mass index; LDL-C, low-density lipoprotein cholesterol; SII, systemic immune-inflammation index; PLR, platelet-to-lymphocyte ratio; SIRI, systemic inflammation response index; LMR, lymphocyte-to-monocyte ratio.

### 3.5 Machine learning and ablation analyses

In the logistic regression (LR) analysis, recursive feature elimination with cross-validation (RFECV) identified LXA4, LTB4, sex, history of cerebral infarction, and diabetes as the optimal feature set. In the linear-kernel support vector machine (SVM) analysis, recursive feature elimination (RFE) selected LXA4, total cholesterol, LDL-C, homocysteine (Hcy), SII, SIRI, sex, history of cerebral infarction, hypertension, and diabetes as the optimal feature set. LXA4 was retained in the optimal feature subset identified by both algorithms.

For the LR model, the best-feature model achieved an AUC of 0.680 (95% CI 0.568–0.790) in the training set and 0.676 (95% CI 0.504–0.826) in the test set. By comparison, the LXA4-only model yielded a test-set AUC of 0.584, and the LTB4-only model yielded a test-set AUC of 0.520. Removal of both LXA4 and LTB4 reduced the test-set AUC of the clinical-feature model to 0.615.

For the SVM model, the integrated model including LXA4 achieved an AUC of 0.681 (95% CI 0.554–0.802) in the training set and 0.659 (95% CI 0.492–0.807) in the test set. In the test set, sensitivity, specificity, and accuracy were 0.718, 0.643, and 0.698, respectively. The SVM model using LXA4 alone yielded a test-set AUC of 0.557, whereas the corresponding control model without LXA4 showed a markedly lower test-set AUC of 0.383.

**Figure 1:**
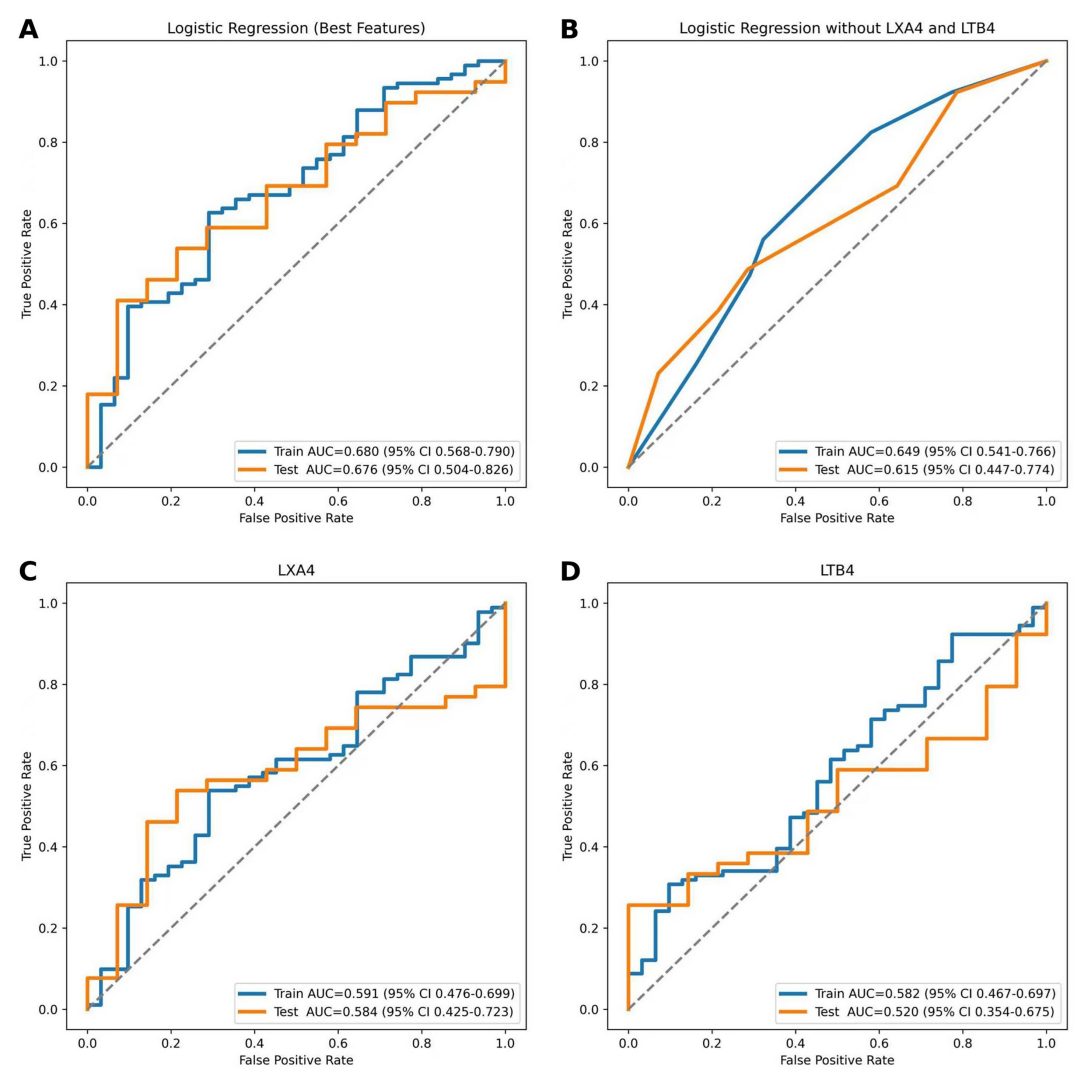
Receiver operating characteristic (ROC) curves of logistic regression models for predicting 3-month functional outcome. **(A)** Best-feature logistic regression model: training AUC = 0.680 (95% CI 0.568–0.790), test AUC = 0.676 (95% CI 0.504–0.826). This model showed the best overall discriminative performance. **(B)** Logistic regression model excluding LXA4 and LTB4: training AUC = 0.649 (95% CI 0.541–0.766), test AUC = 0.615 (95% CI 0.447–0.774). Model performance decreased after removal of LXA4 and LTB4. **(C)** LXA4-only logistic regression model: training AUC = 0.591 (95% CI 0.476–0.699), test AUC = 0.584 (95% CI 0.425–0.723). LXA4 alone showed modest discriminative ability. **(D)** LTB4-only logistic regression model: training AUC = 0.582 (95% CI 0.467–0.697), test AUC = 0.520 (95% CI 0.354–0.675). LTB4 alone showed limited discriminative performance. Blue lines indicate training-set performance, and orange lines indicate test-set performance. The dashed diagonal line indicates chance-level discrimination (AUC = 0.5). Ninety-five percent confidence intervals were estimated by bootstrap resampling. AUC, area under the receiver operating characteristic curve; CI, confidence interval; LXA4, lipoxin A4; LTB4, leukotriene B4.

**Figure 2:**
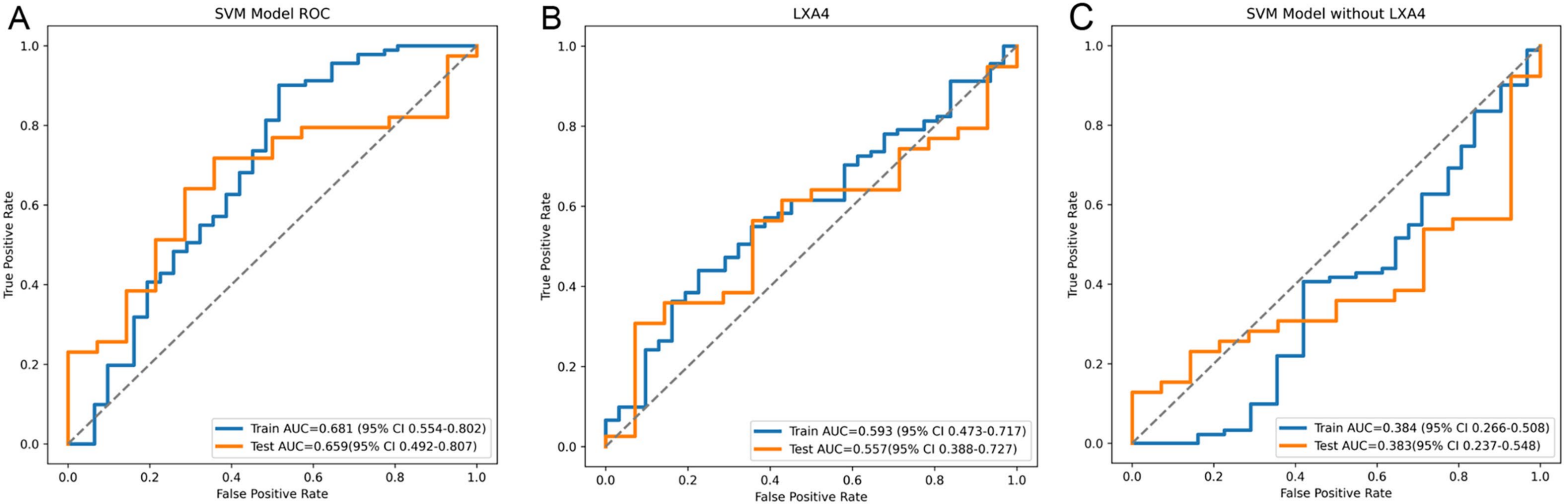
Receiver operating characteristic (ROC) curves of support vector machine (SVM) models for predicting 3-month functional outcome. **(A)** Best-feature SVM model: training AUC = 0.681 (95% CI 0.554–0.802), test AUC = 0.659 (95% CI 0.492–0.807). Training-set sensitivity, specificity, and accuracy were 0.484, 0.742, and 0.549, respectively; the corresponding values in the test set were 0.718, 0.643, and 0.698. This model showed the best overall discriminative performance among the SVM models. **(B)** LXA4-only SVM model: training AUC = 0.593 (95% CI 0.473–0.717), test AUC = 0.557 (95% CI 0.388–0.727). Training-set sensitivity, specificity, and accuracy were 0.363, 0.839, and 0.484, respectively; the corresponding values in the test set were 0.308, 0.929, and 0.472. LXA4 alone showed modest discriminative ability. **(C)** SVM model excluding LXA4: training AUC = 0.384 (95% CI 0.266–0.508), test AUC = 0.383 (95% CI 0.237–0.548). Training-set sensitivity, specificity, and accuracy were 0.000, 0.839, and 0.213, respectively; the corresponding values in the test set were 0.128, 1.000, and 0.358. Removal of LXA4 was associated with a marked decline in model performance, with AUC values falling below the chance level. Blue lines indicate training-set performance and orange lines test-set performance. The dashed diagonal line indicates chance-level discrimination (AUC = 0.5). Ninety-five percent confidence intervals were estimated by bootstrap resampling. AUC, area under the receiver operating characteristic curve; CI, confidence interval; LXA4, lipoxin A4; SVM, support vector machine.

Overall, the ablation analysis suggested that models incorporating LXA4 performed better than both the single-marker models and the corresponding control models without LXA4. The detailed results are shown in Tables 5, 6 and Figures 1, 2.

**Table 5:** Performance of four logistic regression models for predicting 3-month functional outcome.

Model	Training AUC	Test AUC	Sensitivity (train/test)	Specificity (train/test)	Accuracy (train/test)	F1 score (train/test)	MCC (train/test)	Interpretation
Model 1: optimal feature set (LXA4 + LTB4; 5 features)	0.680	0.676	0.626/0.410	0.710/0.929	0.648/0.547	0.726/0.571	0.294/0.320	Best overall-performing model
Model 2: LXA4 alone	0.591	0.584	0.538/0.538	0.710/0.786	0.582/0.604	0.658/0.667	0.216/0.287	Single-marker model based on LXA4
Model 3: LTB4 alone	0.582	0.520	0.308/0.256	0.903/1.000	0.459/0.453	0.459/0.408	0.211/0.289	Single-marker model based on LTB4
Model 4: clinical-feature model excluding LXA4 and LTB4	0.649	0.615	0.824/0.487	0.419/0.714	0.721/0.547	0.815/0.613	0.249/0.179	Reduced performance after removal of lipid mediators

Values are presented as training set/test set. AUC, area under the receiver operating characteristic curve; MCC, Matthews correlation coefficient. Model 1 showed the best overall predictive performance. Removal of LXA4 and LTB4 was associated with reduced model performance.

**Table 6:** Performance of three support vector machine models for predicting 3-month functional outcome.

Model	Training AUC	Test AUC	Sensitivity (train/test)	Specificity (train/test)	Accuracy (train/test)	Interpretation
Model 1: best-feature SVM model including LXA4 (10 features)	0.681	0.659	0.484/0.718	0.742/0.643	0.549/0.698	Best overall-performing model
Model 2: LXA4-only SVM model	0.593	0.557	0.363/0.308	0.839/0.929	0.484/0.472	Single-marker model based on LXA4
Model 3: SVM model excluding LXA4 (9 features)	0.384	0.383	0.000/0.128	0.839/1.000	0.213/0.358	Marked performance deterioration after exclusion of LXA4

Values are presented as training set/test set. AUC, area under the receiver operating characteristic curve. Model 1 showed the best overall predictive performance. Removal of LXA4 was associated with a marked decline in model performance. In Model 3, the AUC was below 0.5, suggesting performance inferior to random classification.

## 4 Discussion

### 4.1 Main findings

The present study investigated the relationship between specialized pro-resolving lipid mediator profiles and functional outcome in patients with LAA stroke and yielded three principal findings. First, in the conventional outcome-group comparison, serum LXA4 levels were higher in patients with a good outcome than in those with a poor outcome, although the between-group difference did not reach statistical significance. This pattern suggests a directional association between LXA4 and functional outcome, but does not support its use as a stand-alone prognostic marker. Second, simple binary stratification according to the presence or absence of T2DM did not reveal significant differences in LXA4, LTB4, or RvD2. In contrast, further stratification within the diabetic subgroup by diabetes duration identified a distinct pattern in patients with a duration of ≥ 5 years, characterized by higher LXA4, higher LTB4, and a lower RvD2/LTB4 ratio; diabetes duration was also positively correlated with both LXA4 and LTB4. Third, in the exploratory machine learning analyses, LXA4 was consistently retained during feature selection across different models, and model performance declined after its removal. Collectively, these findings suggest that stratification by diabetes duration, rather than by diabetes status alone, may better capture heterogeneity in lipid mediator regulation in LAA stroke. They further support the interpretation of LXA4 as a candidate signaling molecule linking the metabolic-inflammatory milieu to stroke outcome, rather than as a marker defined by a strong univariable main effect.

This interpretation requires caution. In the present cohort, the proportion of poor 3-month outcome was lower in the T2DM group than in the non-T2DM group, and the two groups also differed in age structure and smoking exposure. These imbalances suggest the presence of complex baseline confounding. Accordingly, the present findings should not be interpreted as evidence that T2DM exerts a protective effect on outcome after LAA stroke. Rather, they indicate that, within the current sample composition, dichotomous stratification according to diabetes status was insufficient to reliably discriminate differences in lipid mediators or clinical outcome.

With respect to LXA4 itself, only a borderline signal was observed in the conventional statistical comparisons, whereas a more consistent pattern emerged in the multivariable framework. This suggests that the potential value of LXA4 may lie less in univariable statistical significance than in the incremental information it provides when considered together with other clinical and biochemical variables.

This observation is broadly in line with previous clinical evidence. Prior studies have shown that peripheral blood LXA4 levels are lower in patients with post-stroke cognitive impairment than in those without cognitive impairment, and that LXA4 levels correlate positively with cognitive scores, suggesting that reduced LXA4 may be associated with less favorable neurological recovery (16). Although cognitive impairment was not the endpoint in that study, the direction of the association is consistent with the trend observed here, namely that patients with better functional outcome tended to have relatively higher LXA4 levels.

Experimental and review-based evidence likewise supports a biologically plausible role for LXA4 in ischemic stroke. Previous studies have shown that LXA4 and related pathways are associated with attenuation of cerebral edema, reduction in infarct size, and mitigation of neuroinflammation in experimental models of ischemic stroke (15). Mechanistically, LXA4, as a key member of the specialized pro-resolving lipid mediator family, exerts potent anti-inflammatory and pro-resolving actions through binding to its receptor ALX/FPR2. These actions include inhibition of neutrophil chemotaxis and infiltration, promotion of macrophage clearance of apoptotic neutrophils, induction of macrophage polarization from the pro-inflammatory M1 phenotype toward the anti-inflammatory M2 phenotype, and facilitation of the release of neuroprotective factors such as brain-derived neurotrophic factor (21). From this perspective, the directionally favorable association between higher LXA4 levels and better outcome observed in the present study is biologically credible.

Overall, the most important implication of this study is not that LXA4 has already been established as an independent threshold-based prognostic biomarker. Rather, the data suggest that, in LAA stroke, particularly in the setting of long-duration T2DM, alterations in LXA4 and related lipid mediators may be more appropriately understood as part of a broader remodeling of specialized pro-resolving lipid mediator profiles. Within this process, LXA4 may represent a candidate signaling molecule with translational relevance, meriting further evaluation in larger cohorts and in analytical models incorporating more comprehensive clinical covariates.

### 4.2 Diabetes duration-related remodeling of lipid mediator profiles

Compared with simple dichotomous stratification by the presence or absence of T2DM, further stratification within the diabetic subgroup according to diabetes duration revealed clearer differences in lipid mediator profiles. This finding suggests that diabetes duration may more adequately reflect the cumulative impact of chronic metabolic-inflammatory exposure on inflammation-resolution networks than diabetes status alone.

Specifically, patients with a diabetes duration of ≥ 5 years exhibited a combined pattern of higher LXA4 levels, higher LTB4 levels, and a lower RvD2/LTB4 ratio. In parallel, diabetes duration was positively correlated with both LXA4 and LTB4, whereas the RvD2/LTB4 ratio was negatively correlated with both mediators. The overall concordance between the stratified and correlation analyses favors the interpretation of lipid mediator profile remodeling rather than isolated alterations in individual mediators.

Taken together, these findings suggest that long-duration T2DM may be associated with a broader reconfiguration of inflammatory regulation, rather than a simple linear change in a single lipid mediator. Mechanistic interpretation of this pattern, however, is limited by the cross-sectional, observational design of the present study. The proposed notion of coexisting compensatory pro-resolving activation and persistent pro-inflammatory imbalance remains speculative and is derived from experimental and indirect evidence rather than from data generated in this cohort. Causal inference regarding the dynamic evolution of inflammation-resolution networks is not supported by a single peripheral blood measurement.

This interpretation is broadly consistent with previous clinical evidence. Earlier studies have shown that the RvD2/LTB4 ratio is lower in patients with diabetes associated ischemic stroke than in non-diabetic stroke patients, and that a lower ratio is also associated with poorer outcome, suggesting greater vulnerability of the pro inflammatory/pro-resolving balance in the diabetic setting (17). In addition, among patients with acute ischemic stroke undergoing endovascular therapy, postoperative levels of LXA4, LTB4, and their relative ratios have been associated with early neurological deterioration and 90-day functional outcome (22). Translational studies on diabetes-related acute ischemic stroke have further suggested that inflammation resolution may be impaired under diabetic conditions, and that RvD2-related interventions can partially ameliorate this state of insufficient resolution in experimental settings (23). These observations provide indirect support for the present finding that the RvD2/LTB4 ratio was reduced in the long-duration group despite the absence of a parallel decline in absolute mediator levels. Of note, the T2DM and non-T2DM groups showed notable baseline imbalances in age distribution and smoking prevalence, which represent potential confounders. The subgroup analyses by diabetes duration involved small samples (*n* = 27 and *n* = 25) and should be interpreted with caution as exploratory findings subject to residual confounding and limited statistical power.

Accordingly, the increase in LXA4 observed in the present study should not be interpreted simply as evidence of adequately restored inflammation resolution. Rather, it should be viewed in the context of the coordinated changes in LXA4, LTB4, and the RvD2/LTB4 ratio. From this perspective, patients with long-duration T2DM may be more likely to exhibit reduced quality of inflammatory regulation and imbalance within the lipid mediator network, rather than a simple linear abnormality in a single mediator.

### 4.3 Candidate value of LXA4 in the machine learning analyses

In the present study, machine learning was used primarily to assess the potential informational contribution of LXA4 to outcome discrimination across different algorithmic frameworks, rather than to generate evidence for a clinically deployable prediction tool (24).

Feature-selection results showed that the RFECV-based logistic regression model retained five variables in the optimal feature set, namely LXA4, LTB4, sex, history of cerebral infarction, and diabetes. By contrast, the RFE-based linear-kernel support vector machine (SVM) model retained 10 variables: LXA4, total cholesterol, LDL-C, homocysteine, SII, SIRI, sex, history of cerebral infarction, hypertension, and diabetes. Although the final feature composition differed between the two models, LXA4 was consistently retained in both optimal subsets, suggesting that its informational value was not specific to a single modeling strategy.

This pattern was also reflected in model performance. In the logistic regression analysis, the combined model incorporating LXA4, LTB4, sex, history of cerebral infarction, and diabetes achieved a test-set AUC of 0.676, whereas the LXA4-only model, the LTB4-only model, and the clinical model excluding inflammatory lipid mediators all showed lower discriminative performance. A similar pattern was observed in the linear-kernel SVM analysis. The integrated model including LXA4 achieved a test-set AUC of 0.659, whereas removal of LXA4 reduced the AUC to 0.383, indicating a marked deterioration in discrimination after exclusion of this feature.

Taken together, the feature-selection and ablation results suggest that LXA4 is better interpreted as a candidate feature with stable retention and incremental informational value in multivariable models, rather than as a marker defined primarily by a strong univariable effect. This interpretation is consistent with the overall pattern of the present study: although LXA4 showed only a trend-level difference in conventional group comparisons, it exhibited relatively consistent directional information across diabetes duration-based stratification, correlation analyses, and machine learning analyses.

These findings should, however, be interpreted cautiously. The present study was based on a single-center cohort with a limited sample size, and model evaluation relied mainly on internal training-test splitting without independent external validation. The machine learning results should therefore be regarded as exploratory evidence regarding candidate feature contribution rather than as proof of model generalizability. According to current reporting and appraisal frameworks for clinical prediction models, performance metrics derived from internal validation mainly reflect discrimination within the available sample, whereas model transportability and practical applicability require confirmation in independent external datasets (24, 25).

Accordingly, the principal value of the machine learning analyses in this study lies in providing an additional analytical perspective supporting the potential relevance of LXA4 as a candidate signaling molecule. More broadly, the findings suggest that joint analysis of specialized pro-resolving lipid mediators and clinical variables may allow a more refined characterization of prognostic heterogeneity in patients with LAA stroke.

### 4.4 Clinical implications and translational relevance

The present findings suggest that, in patients with LAA stroke, diabetes duration may provide more clinically relevant information than diabetes status alone for identifying subgroups with distinct patterns of inflammatory regulation. This inference is supported by the observation that no significant differences in LXA4, LTB4, or RvD2 were detected with simple dichotomous stratification by T2DM status, whereas further stratification by diabetes duration revealed a combined pattern in the long-duration subgroup characterized by higher LXA4, higher LTB4, and a lower RvD2/LTB4 ratio. These results indicate that treating diabetes merely as a binary comorbidity may be insufficient to capture heterogeneity in the metabolic-inflammatory milieu, whereas incorporation of diabetes duration may allow a more refined characterization of inflammatory regulatory status.

At the same time, the present data do not support interpreting LXA4 as an independent prognostic marker with a fixed threshold effect. Rather, its potential translational relevance may lie in the incremental information it provides when considered jointly with LTB4, the RvD2/LTB4 ratio, and baseline clinical variables. The machine learning analyses further support this view, suggesting that combined evaluation of specialized pro-resolving lipid mediators and clinical variables may better characterize prognostic heterogeneity in LAA stroke, particularly in patients with long-duration T2DM, in whom metabolic-inflammatory dysregulation may be more pronounced.

This potential value should, however, be interpreted appropriately. The contribution of these markers lies primarily in refining clinical stratification rather than in providing a stand-alone tool capable of replacing established stroke severity assessment, neuroimaging evaluation, or standardized follow-up systems. According to current principles for the evaluation of clinical prediction models, performance metrics derived from internal training-test splitting primarily reflect discrimination within the available dataset and are insufficient to establish generalizability or immediate clinical utility. Accordingly, the most appropriate translational interpretation of the present study is that it provides a rationale for future multicenter validation, composite biomarker integration, and more advanced risk stratification research, rather than a directly implementable clinical decision tool.

It should also be acknowledged that the present study did not formally evaluate whether lipid mediators add predictive value beyond established clinical variables. A rigorous assessment of incremental prognostic value would require a reference model built from key prognostic covariates such as stroke severity (NIHSS score), infarct volume, and acute treatment status, against which models incorporating lipid mediators could be compared. Because these variables were not systematically collected in the present retrospective dataset, such a comparison could not be performed. Accordingly, the clinical relevance of the lipid mediators examined here remains to be established in future studies incorporating comprehensive covariate adjustment (26).

### 4.5 Limitations and future directions

Several limitations should be considered when interpreting the present findings. This study is explicitly exploratory, and all findings should be interpreted accordingly.

First, this was a single-center retrospective study with a limited sample size, and the diabetic subgroup (*n* = 52) became relatively small after stratification by disease duration. This may have reduced statistical power for some comparisons and may also have affected the robustness of the machine learning analyses and the external validity of the findings. Second, lipid mediators were measured at only a single time point within 72 h after stroke onset, precluding assessment of the dynamic evolution of inflammation-resolution networks from the acute to the recovery phase. Third, important variables related to stroke severity and treatment, including baseline NIHSS score, infarct volume, and reperfusion therapy, were not systematically incorporated into the analysis. This represents a major limitation: without adjustment for established stroke outcome predictors, it is not possible to assess whether lipid mediators are independently associated with prognosis. No claim of independent prognostic value for LXA4 or related indicators can be made from this dataset. In addition, prior and in-hospital medication exposure, including statins, aspirin, and ACEI/ARB agents, was not systematically controlled for, although these factors may affect inflammatory markers, lipid metabolism, and clinical outcome. Finally, the machine learning models were developed and evaluated mainly through internal training-test splitting without independent external validation. The train/test split further reduces effective sample size, and the modest AUC values (approximately 0.65–0.68) underscore the limited performance. Feature selection was performed within a small dataset, increasing the risk of overfitting. These results should therefore be regarded as hypothesis-generating only, and the interpretation that LXA4 is an important predictor should not be overstated.

Future studies should validate these findings in multicenter cohorts, larger samples, and independent external datasets, with particular attention to the reproducibility of LXA4, LTB4, and the RvD2/LTB4 ratio across different populations and clinical settings. Longitudinal sampling designs with serial measurements of LXA4, LTB4, RvD2, and their relative ratios will also be needed to better define the temporal evolution of specialized pro-resolving lipid mediator profiles during both the acute and recovery phases of stroke. If future studies further incorporate stroke severity, treatment exposure, and broader lipidomic information, and integrate these data within composite biomarker frameworks, the stratification approach proposed here may evolve into a more clinically interpretable and potentially useful strategy for individualized risk stratification.

## 5 Conclusion

The present study demonstrates diabetes duration-related remodeling of specialized pro-resolving lipid mediator profiles in patients with LAA stroke. Specifically, long-duration T2DM was associated with higher LXA4 and LTB4 levels together with a lower RvD2/LTB4 ratio.

Compared with simple dichotomous stratification according to the presence or absence of T2DM, stratification by diabetes duration revealed clearer differences in lipid mediator profiles, suggesting that disease duration may better reflect alterations in inflammation-regulatory networks within a metabolic-inflammatory milieu.

Although LXA4 showed only a trend-level difference in conventional outcome-group comparisons, it displayed a relatively consistent directional pattern across diabetes duration-based stratification, correlation analyses, and machine learning analyses. These findings suggest that LXA4 may represent a candidate signaling molecule that may be relevant to functional outcome after stroke; however, these findings are exploratory, the association with outcome did not reach statistical significance, and no independent prognostic value has been demonstrated.

Overall, the main implication of this study is that stratification by diabetesduration may help identify metabolic-inflammatory heterogeneity in patients with LAA stroke and may provide a rationale for future research. Given the exploratory nature of the present findings and the absence of key clinical confounders (stroke severity, acute treatment), these results should not be interpreted as establishing clinical prognostic utility stratification strategies. Further validation in multicenter studies, larger cohorts with comprehensive covariate adjustment, and independent external datasets is required before any clinical conclusions can be drawn.

## Data Availability

The original contributions presented in the study are included in the article/Supplementary material, further inquiries can be directed to the corresponding author.
